# Superior improvement in dynamic response of liquid crystal lens using organic and inorganic nanocomposite

**DOI:** 10.1038/s41598-021-96991-4

**Published:** 2021-08-30

**Authors:** Che Ju Hsu, Bhupendra Pratap Singh, Pravinraj Selvaraj, Mareena Antony, Rajiv Manohar, Chi Yen Huang

**Affiliations:** 1 Graduate Institute of Photonics, National Changhua University of Education, Changhua, 500 Taiwan; 2 Liquid Crystal Research Lab, Physics Department, University of Lucknow, Lucknow, 226007 India; 3 Department of Physics, National Changhua University of Education, Changhua, 500 Taiwan

**Keywords:** Engineering, Materials science, Nanoscience and technology, Optics and photonics

## Abstract

In this study, the response time of a 4 mm-aperture hole-patterned liquid crystal (HLC) lens has been significantly improved with doping of N-benzyl-2-methyl-4-nitroaniline (BNA) and rutile titanium dioxide nanoparticle (TiO_2_ NP) nanocomposite. The proposed HLC lens provides the focus and defocus times that are 8.5× and 14× faster than the pristine HLC lens, respectively. Meanwhile, the focus and defocus times of the proposed HLC lens reach the order of millisecond. Result shows that the synergistic effect of BNA and TiO_2_ NP induces a 78% decrement in the viscosity of pristine LC mixture that significantly shortens the focus and defocus times of HLC lens. The remarkable decrement in viscosity is mainly attributed to spontaneous polarization electric fields from the permanent dipole moments of the additives. Besides, the strengthened electric field surrounding TiO_2_ NP assists in decreasing the focus time of HLC lens. The focus and defocus times of HLC lens are related to the wavefront (or phase profile) bending speed. The time-dependent phase profiles of the HLC lenses with various viscosities are calculated. This result shows the decrease in wavefront bending time is not simply proportional to viscosity decrement. Furthermore, the proposed HLC lens emerges a larger tunable focus capability within smaller voltage interval than the pristine HLC lens.

## Introduction

Liquid crystal (LC) lenses have attracted great attention in photonic fields due to their low weight and cost and electrically-controllable focal length^1^. Several approaches had been presented to develop large-aperture LC lenses, such as the collocation of spherical glass and LC layer^2^, dielectric dividing principle^3^, polymer network LCs^4^, cholesteric LCs^5^, smectic LCs^6^, multiple ring electrodes^7^, and hole-patterned electrode^8^. Among the above-mentioned manners, the hole-patterned LC (HLC) lenses had the advantages of easy construction, simple addressing scheme, and broad tunable focal range. In HLC lenses, an LC layer was sandwiched between hole-patterned and planar electrodes. When a voltage was subjected to the lens cell, the hole-patterned electrode created an axially symmetric nonuniform electric field and aligned the LCs to induce a refractive index gradient in the radial direction. The incident light was converged after passing through the lens cell. However, the effect of weak fringing electric field and the intrinsic high rotational viscosity of LC mixture caused that large aperture HLC lens (aperture size > 4 mm) had a slow dynamic response in the order of several seconds or ten seconds. For example, the focus time of the 7 mm aperture HLC lens was ~ 50 s, which was further reduced to ~ 20 s by applying an extra horizontal field^9^; the defocus time of a 4 mm aperture HLC lens introduced with a pair of ring and pie electrodes was ~ 12 s, which was further shortened to ~ 5 s by the dual-frequency driving scheme^10^. The switching speeds of the HLC lenses were not fast enough for practical applications. Adopting the nematic LCs with low viscosity was a straightforward method to shorten the response time of LC devices^11,12^. Other manners, such as polymer-stabilized LCs^13^ and nanoparticle (NP) dispersion^14^, were also reported to improve the dynamic response of LC devices. In our recent studies, the inorganic rutile titanium dioxide (TiO_2_) NP doping was used to fabricate the 4 mm aperture HLC lens^15^. The TiO_2_ NP-doped HLC lens had the focus and defocus times, which were 3.6× and 2.3× faster than the pristine HLC lens, respectively. Moreover, the TiO_2_ NP-doped HLC lens provided a wider tunable focal length range with the lower operation voltage than the pristine HLC lens. Subsequently, an HLC lens was fabricated with doping of organic N-benzyl-2-methyl-4-nitroaniline (BNA). The BNA-doped HLC lens had the focus and defocus times, which were 3.0× and 6.7× faster than the pristine HLC lens, respectively^16^, but without any improvement in the operating voltage of lens. In general, the focus and defocus times of the HLC lenses still exceed 1 s.

Based on the above results, the BNA and rutile TiO_2_ NP (BR) composite in this study has been used to fabricate a fast response HLC lens for the first time. The BR’s synergetic effects on the electro-optical properties of HLC lens were investigated. The optical interference fringes and focal lengths of the pristine and BR composite-doped HLC lenses were measured to obtain the phase distribution and compare the tunable focusing capability. The BR composite-doped HLC lens provides the wider focal range within smaller voltage interval than the pristine HLC lens. The response time of the HLC lenses was obtained by measuring the focus and defocus transient intensities. In comparison with previous manners^15,16^, BR composite doping further enhances the response speed of HLC lens; accordingly, the BR composite-doped HLC lens has the short focus and defocus times, which are 8.5× and 14× faster than the pristine HLC lens, respectively. Meanwhile, the focus and defocus times of the BR composite-doped HLC lens reach the order of milliseconds. The mechanisms for the fast focus and defocus responses with BR composite doping have been explained in detail.

## Methods

**Figure 1:**
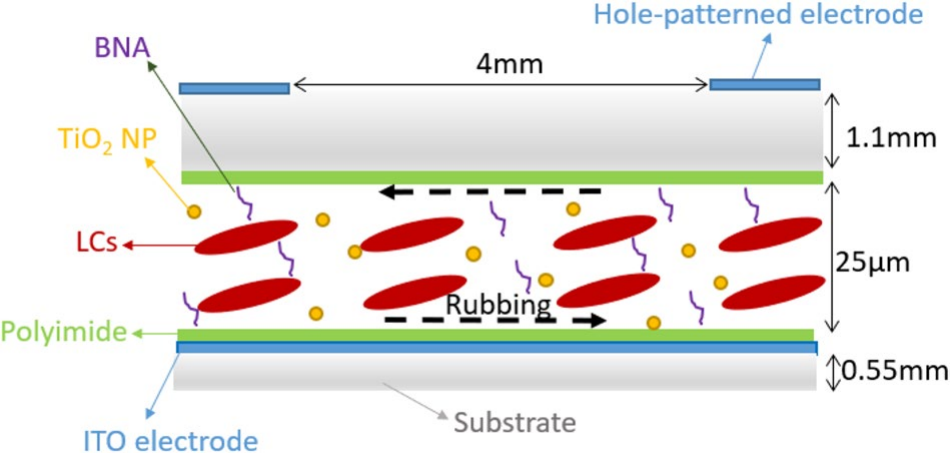
Diagram of the BR composite-doped HLC lens structure.

In Fig. 1, the BR composite-doped HLC lens cell used in the experiment was composed of two indium–tin–oxide (ITO) glass substrates (Chipset Technology, Miaoli, Taiwan). The thickness of top and bottom glass substrates was 1.1 and 0.55 mm, respectively. The ITO surface of the top glass substrate was etched with a circular-hole pattern of 4 mm diameter by the photolithography process. The inner surfaces of both substrates were coated with a homogeneous polyimide (PI) AL 1426 CA (Daily Polymer Kaohsiung, Taiwan) and rubbed in antiparallel directions^15^. The cell gap of lens cell was controlled at ~ 40 μm thick by Mylar spacer. An LC mixture was prepared with the nematic LC E7 (Daily Polymer Kaohsiung, Taiwan), inorganic rutile TiO_2_ NPs (SkySpring Nanomaterials, Inc., USA), and organic BNA (Seedchem Company Pty. Ltd, Australia) at the concentration ratio of 96.5:0.5:3. The concentration ratios of TiO_2_ NP and BNA were decided according to our previous works^15,16^. The supplier information indicated that LC E7 had a dielectric anisotropy *Δε* of 14.5, birefringence *Δn* of 0.216 at room temperature, and elastic constants *K*_*11*_, *K*_*22*_, and *K*_*33*_ of 11.1, 5.9, and 17.1 pN, respectively; the average diameter of the rutile TiO_2_ NP was ~ 20 nm^14^. Moreover, the rutile TiO_2_ NPs were treated with surface silane-coating and dissolved in ethanol for well dispersion in LCs. The LC mixture was first heated up to isotropic phase and then filled in the HLC lens cell by the capillary action. The ethanol was evaporated during heating. After filling, the LC mixture was cooled down to nematic phase. The HLC lens filled with pristine E7 was also fabricated for comparison and defined as the pristine HLC lens.

The homogeneously pristine, rutile TiO_2_ NP-doped, BNA-doped, and BR composite-doped LC cells were also fabricated to explain the effects of the BR composite in LCs. The LC cells were treated with antiparallel rubbing, and their cell gaps were controlled at 40 μm thick. Here, the BNA and TiO_2_ NP concentrations were set at 3 and 0.5 wt%, respectively. The rotational viscosity (*γ*) of the LC mixture was experimentally determined by transient-current method^17^. The polarizing optical microscope (POM) (DM EP, LEICA, Germany) images of the LC cell with various voltages were measured to determine the threshold voltage (*V*_*th*_) of the cell. *V*_*th*_ was defined as the voltage at which the color of the POM image began to change, thereby indicating the initial distortion of LCs in the middle of the cell^18^. The response time of the LC cell was measured by the following setup^19^. A He–Ne laser with 632 nm wavelength was used as incident light, and the LC cell was placed between a pair of crossed polarizers. The rubbing direction of the LC cell had an angle of 45° with respect to the transmission axes of the polarizers. A photodetector connected to the oscilloscope was placed behind the analyzer to record the transient transmission. The rise (fall) time of the LC cell was defined as the time required for the transmission to change from 90 to 10% (10% to 90%) of the maximum transmission when the LC cell was turned on from 10.8 to 60.0 V (turned off from 60.0 to 10.8 V).

In practical applications, the LC lens acted as an optical component to converge or diverge incident light. As a result, the time-dependent intensity of spot at the focal plane was suitable to determine the response time of the HLC lens^9,20–22^, instead of the transient transmission under the crossed polarizers (to obtain the response time of the LC cell with uniform planar electrodes). The setup was described as follows: the HLC lens cell was placed behind a polarizer, which transmission axis was parallel to the rubbing direction of the lens cell. The expanded He–Ne laser was incident normally through the polarizer and lens cell. The transient intensities were then recorded using a photodetector located at the focal length of the HLC lens. The focus (defocus) time was defined as the time during which the intensity of the HLC lens reached stable when the supplied voltage (100 V) was suddenly turned on (off). With the measurement method, the response speed of the BR composite-doped HLC lens can be compared with those of the HLC lenses demonstrated in our previous works^15,16^. The measurement in focus and defocus times of HLC lens evaluate the wavefront bending characteristics, but that in rise and fall times of LC cell evaluate the transmissions.

## Results and discussion

**Figure 2:**
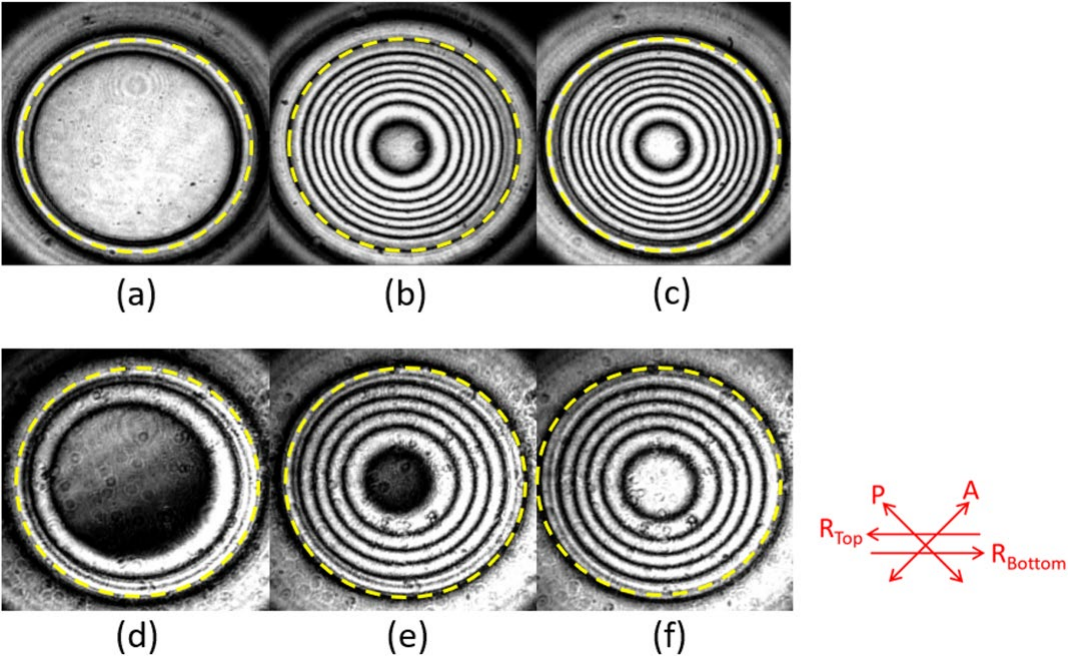
Interference fringes of the pristine HLC lens addressed at (**a**) 30 V, (**b**) 80 V, and (**c**) 100 V; interference fringes of the BR composite-doped HLC lens addressed at (**d**) 30 V, (**e**) 70 V, and (**f**) 80 V. The yellow dashed circle indicates the AH diameter; P and A indicate the transmission axes of polarizer and analyzer, respectively; R_Top_ and R_Bottom_ indicate the rubbing directions on the surfaces of the top and bottom substrates, respectively.

The optical interference fringes of the pristine and BR composite-doped HLC lenses at various voltages were obtained using following setup^15^: HLC lens cell was placed between a pair of crossed polarizers which had the transmission axes of 45° with respect to the rubbing direction of the lens cell. An expanded 632.8 nm-wavelength He–Ne laser was incident normally through the polarizers and lens cell. The interference fringes were recorded with a charge-coupled device (CCD) camera located behind the analyzer. The neighboring bright (dark) fringes indicated a 2π phase difference. The focal length (*f*) of HLC lens was related to the number (*N*) of interference fringes with an appropriate spatial distribution, according to the following formula^23^:

1 $$ {\text{f}} = \frac{{r^{2} }}{2N\lambda }, $$

where *r* is the radius of aperture hole (AH), and λ denotes the wavelength of the incident light. 1/*f* is defined as lens power. In Fig. 2a,d, at low voltage (30 V), the interference fringes gather near the AH periphery, and the center of fringes slightly shifts due to the pretilt angle of LCs. The fringes of the pristine and BR composite-doped HLC lenses distribute throughout the entire AH at 80 and 70 V (Fig. 2b,e), respectively, from which the fringing electric field has reached the AH center. The pristine and BR composite-doped HLC lenses have maximum fringe numbers at 100 and 80 V (Fig. 2c,f), respectively. The composite additive decreases the fringe number and associated lens power of the HLC lens due to the decreased *Δn* of the LC mixture by BNA additive.

**Figure 3:**
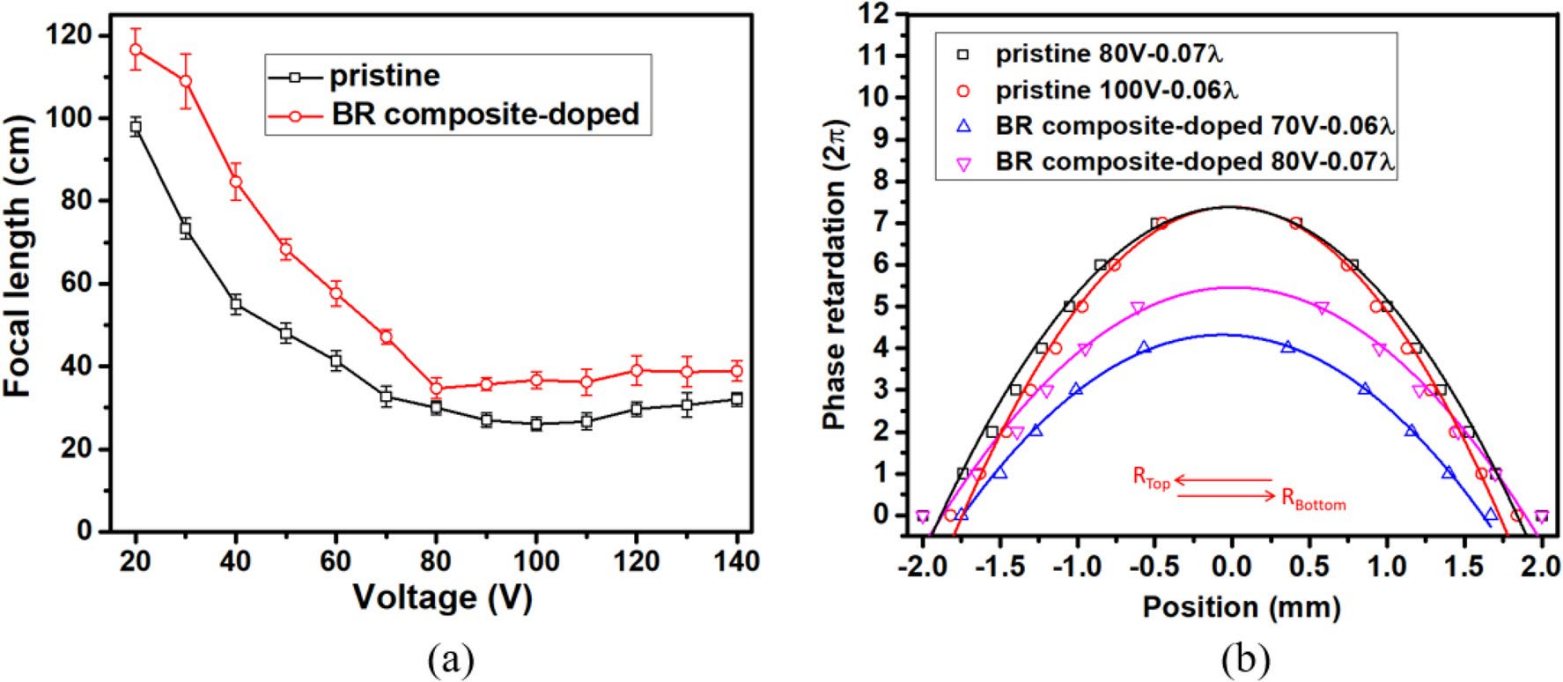
(**a**) Voltage-dependent focal lengths and (**b**) phase retardations of the HLC lenses at various voltages.

The voltage-dependent focal lengths of the HLC lenses were measured using the fringe measurement setup, where the analyzer was removed and the transmission axis of polarizer was adjusted parallel to the rubbing direction of the lens cell. The distance between the LC lens and focusing point was defined as focal length^15^. Figure 3a shows that the minimum focal lengths of the pristine and BR composite-doped HLC lenses are ~ 26.0 and 34.6 cm at 100 and 80 V, respectively. The difference in focal lengths could be attributed to the composite additive decreases the lens power of HLC lens. The focal lengths of pristine and BR composite-doped HLC lenses can be smoothly controlled at 97.9–26.0 cm within 20–100 V and 116.6–34.6 cm within 20–80 V, respectively. As the voltage is below 20 V, the focal lengths of the HLC lenses are too long so that they are ignored. The BR composite-doped HLC lens provides a wider focal range within smaller voltage interval than the pristine HLC lens. The focal length *f* of the pristine HLC lens can be estimated ~ 23 cm by using *r* = 2 mm, *Δn* = 0.216, and d = 40 μm in Eq. (1). The measured minimum focal length ~ 26 cm, close to the estimated *f*. The high operation voltage of HLC lens can be further reduced with application of a high resistivity film for practical application^24–26^.

The interference fringes shown in Fig. 2b,c,e,f were adopted to plot the spatial phase distributions of the HLC lenses^27^, as shown in Fig. 3b. The symbol and solid line indicate the dark fringe position and parabolic fitting curve, respectively. The root mean square (RMS) error of the plotted phase distribution from the ideal parabolic curve was then estimated to examine the wavefront aberration of the HLC lens^27^. As RMS error was less than 1/4 λ, the HLC lens was feasible for ophthalmic applications^28^. In HLC lens, the ratio between AH diameter (D) and glass dielectric thickness (t) was related to the RMS error. The optimum D/t for small HLC lenses (D ~ 1–2 mm) was 2 and that for larger HLC lenses tended toward 3^29^. In this study, the HLC lens had D/t ~ 3.6 but still preserved a low RMS error around 0.07 λ, as shown in Fig. 3b. This result was also confirmed by commercial software LCD master 3D.

**Figure 4:**
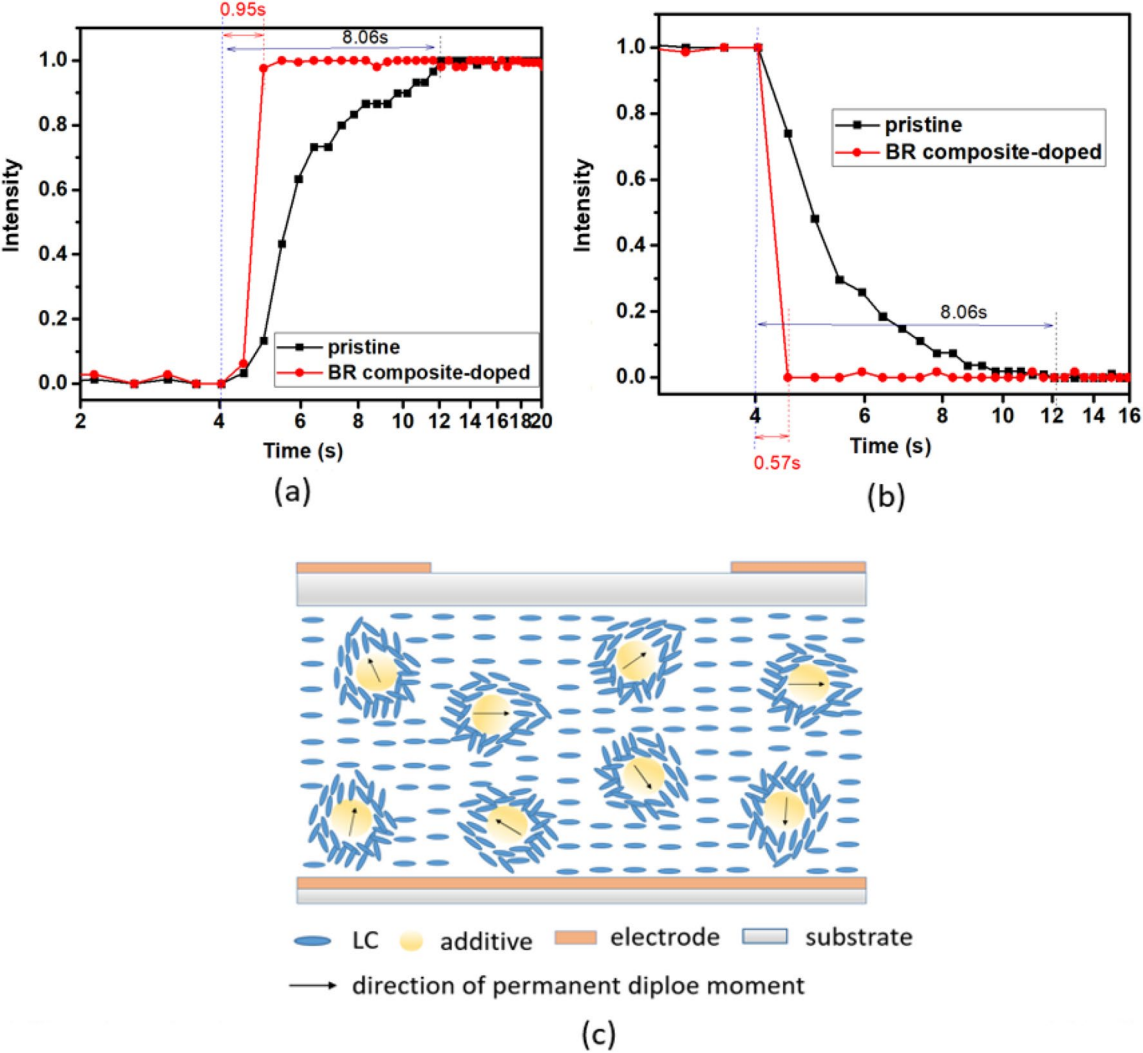
Time-dependent intensities of spots at focal planes when the HLC lenses are turned (**a**) on and (**b**) off; schema of (**c**) SPEFs on the HLC lens.

Figure 4a,b show that the BR composite-doped HLC lens has a focus time that is 8.5 times faster than the pristine HLC lens; meanwhile, the BR composite-doped HLC lens has a defocus time that is 14 times faster than the pristine HLC lens. The fast focus and defocus responses of the BR composite-doped HLC lens are due to the decreased viscosity. When the HLC lens is suddenly supplied with voltage (turned on), the electric field at the AH center almost remains zero or weak, which indicates that the rotation of LCs in the AH center is mainly due to the reorientation process that minimizes the free energy^30^. The rotation of LCs at the AH center follows after the rotation of LCs at the AH periphery. When the supplied voltage suddenly declines (turned off), the LCs at the AH center quickly rewind due to the low tilt angle, and the rotation of LCs at the AH periphery follows after the rotation of the LCs at the AH center by the reorientation process. The decreased viscosity by the BR composite doping shortens the time required for reorientation process and hence the focus and defocus times of HLC lens.

The decreased viscosity is attributed to the coupling interactions between the LC molecule and the permanent dipole moments of the additives. BNA is a polar molecule^31^, and it has a permanent dipole moment because the electron donating and electron accepting groups are located at separate ends of the molecule^32^. Similarly, TiO_2_ NP also has a large permanent dipole moment because of the asymmetric distribution of titanium and oxygen ions at the surface^33,34^. If we consider the additives (BNA and rutile TiO_2_ NP) as dipoles, then the directions of the permanent dipole moments surrounding the additives (local regions) could be different from the director of LCs. The generated local electric fields from the permanent dipole moments are defined as the spontaneous polarization electric field (SPEF)^35^. In Fig. 4c, the SPEF directions of the additives are random under the absence of applied field. The LCs near the local regions orient along the SPEF directions; however, other LCs still align parallel to the cell substrate. Consequently, the additive’s SPEF weakens the dipole–dipole interaction between the LC molecules and hence decreases the viscosity^36^. Moreover, the synergistically intermolecular interactions between the BNA and the TiO_2_ NP strengthens the SPEF that are beneficial in decreasing the viscosity of the LC mixture.

Besides the viscosity, the strengthened electric field surrounding TiO_2_ NP also contributes to the fast focus response of the BR composite-doped HLC lens. The insulating TiO_2_ NP is considered as a high dielectric sphere, which strengthens the electric field on the top and bottom regions of the dielectric sphere along the applied field direction, consequently assisting in the rotation of LCs surrounding the dielectric sphere and decreasing the focus time of the HLC lens^14^.

**Figure 5:**
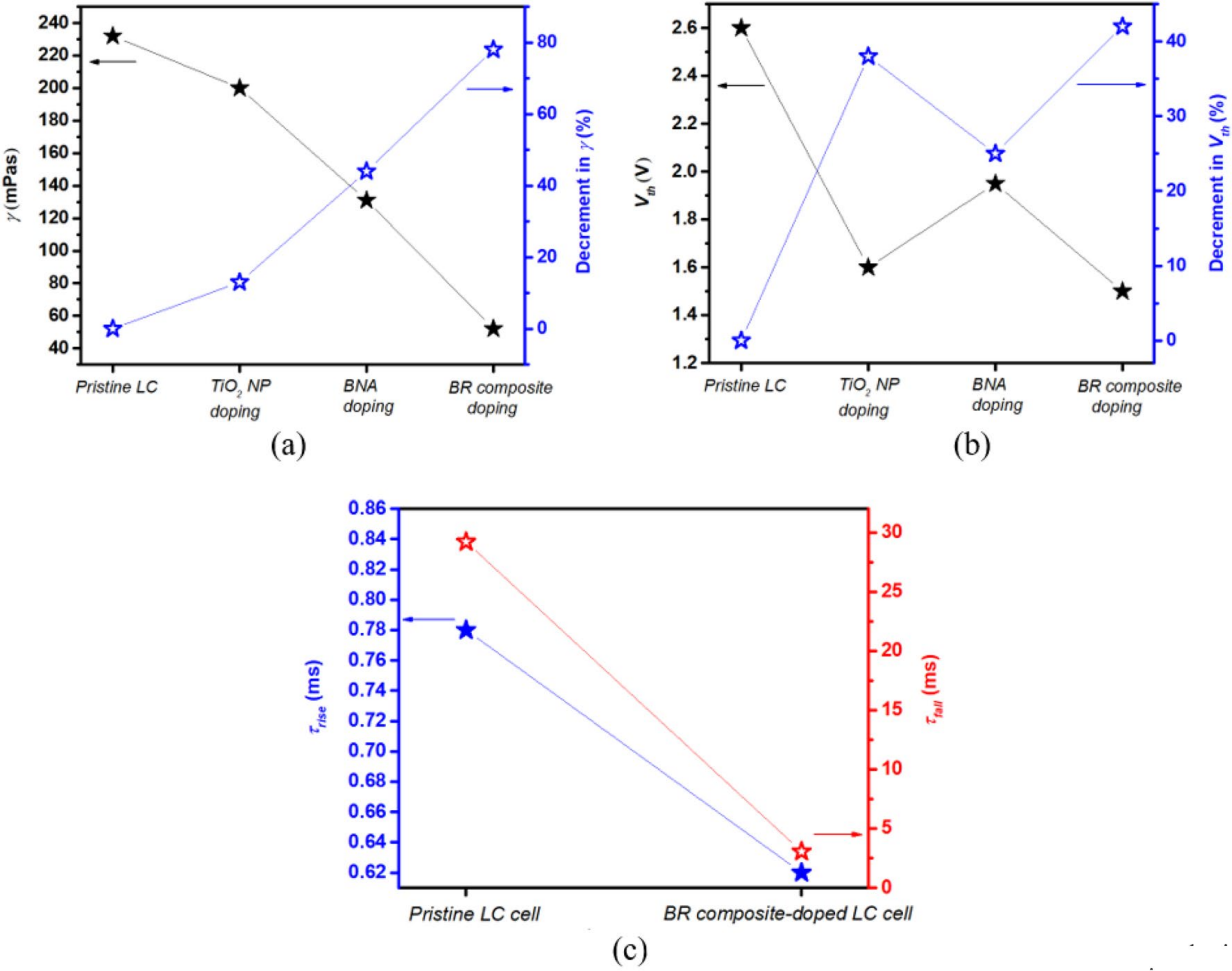
(**a**) *γ* and (**b**) *V*_*th*_ of the LC cells by pristine LC, TiO_2_ NP doping, BNA doping, and BR composite doping. (**c**) Response time of the pristine and BR composite-doped LC cells.

BR’s contributions to decrease *γ* of the LC mixture have been analyzed using the homogenous LC cells. Figure 5a reveals that TiO_2_ NP and BNA dopings induce the 13% and 44% decrements in *γ*, respectively. BR composite doping induces 78% decrement in *γ*, which exceeds the summation of individual contributions of TiO_2_ NP and BNA dopings, thus indicating the decreased *γ* is attributed to BR’s synergistic effect. The rise (*τ*_*rise*_) and fall (*τ*_*fall*_) times of the LC cell are related to *γ* as^37^

2 $$ \tau_{o} = \frac{{\gamma d^{2} }}{{K_{11} \pi^{2} }}, $$

3 $$ \tau_{rise} = \frac{{\tau_{o} }}{{\left| {\left( {\frac{{V_{app} }}{{V_{th} }}} \right)^{2} - 1} \right|}}, $$

4 $$ \tau_{fall} = \frac{{\tau_{o} }}{{\left| {\left( {\frac{{V_{bias} }}{{V_{th} }}} \right)^{2} - 1} \right|}}, $$

where *V*_*app*_ is the applied voltages of cell, *V*_*bias*_ is the bias voltage, and *d* is the cell thickness. *τ*_*0*_ is the relaxation time constant when the LC cell is turned off from *V*_*app*_ that is slightly higher than *V*_*th*_. Equations (2)–(4) indicate that the decreased *γ* and *V*_*th*_ decrease *τ*_*rise*_ and *τ*_*fall*_. In Fig. 5b, the decrement (42%) in *V*_*th*_ with BR composite doping is close to that (38%) in *V*_*th*_ with TiO_2_ NP doping, thus indicating that the decreased *V*_*th*_ with BR composite doping is mainly attributed to TiO_2_ NP component because of the strengthened electric field surrounding the NP. Accordingly, the BR composite-doped LC cell has *τ*_*rise*_ and *τ*_*fall*_ that are ~ 1.25 and 9 times faster than the pristine LC cell, respectively, as shown in Fig. 5c.

**Figure 6:**
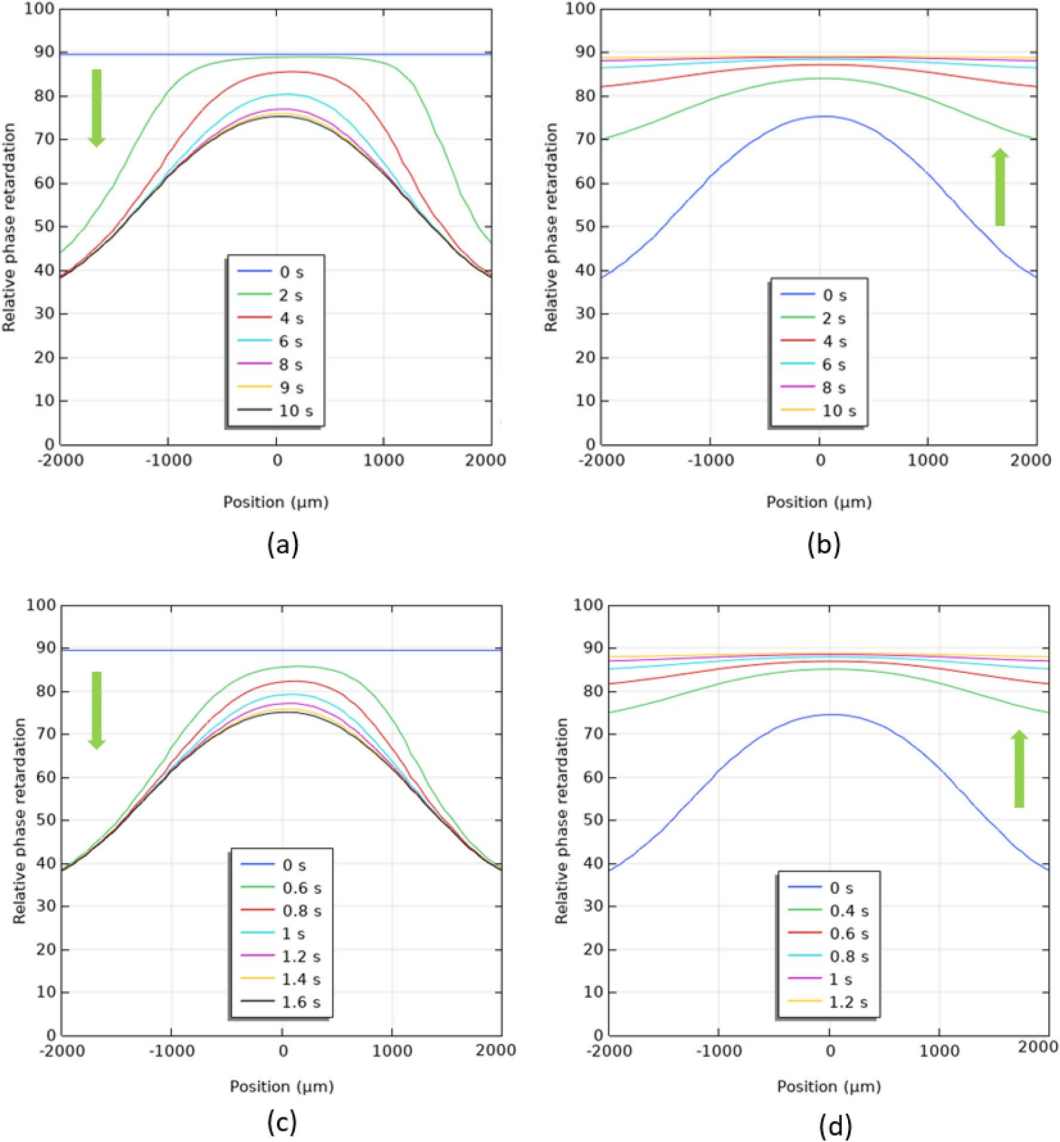
Calculated time-dependent phase (wavefront) conversions in (**a**) voltage turn-on and (**b**) voltage turn-off processes for γ_235_-HLC lens; calculated time-dependent phase (wavefront) conversions in (**c**) voltage turn-on and (**d**) voltage turn-off processes for γ_45_-HLC lens.

The dynamic responses of HLC lenses with various viscosities have been calculated with finite element method software COMSOL. Here the HLC lenses with *γ* = 235 mPas and *γ* = 45 mPas are defined as *γ*_235_- and *γ*_45_-HLC lenses, respectively. The focus (defocus) time is the required time during which planar (parabolic) wavefront of the incident light converts to stable parabolic (planar) wavefront. As shown in Fig. 6, both focus and defocus times of *γ*_235_-HLC lens are 10 s. The focus and defocus times of *γ*_45_-HLC lens are 1.6 s and 1.2 s, respectively. The *γ*_45_-HLC lens has the focus and defocus times that are ~ 6.3× and 8.3× faster than *γ*_235_-HLC lens, respectively. Notably, the 8.3× fast defocus time is not proportional to the viscosity decrement (5×), as predicted from Eq. (2). In addition, the measured focus and defocus times of the pristine and BR composite-doped HLC lenses (Fig. 4a,b) are smaller than the calculated focus and defocus times of *γ*_235_- and *γ*_45_-HLC lenses (Table 1), because the active area of photodetector causes the measured intensity at the focal plane reaches stable earlier than the calculated phase (wavefront) conversion time in Fig. 6.

**Table 1:** Calculated focus and defocus times of the HLC lenses.

	Focus time (s)	Defocus time (s)
γ_235_-HLC lens	10	10
γ_45_-HLC lens	1.6	1.2

In the homogeneous LC cell with planar electrodes, because the exerted voltage is strong, the response time obtained from transmission change is determined by *V*_*app*_, *V*_*bias*_ and viscosity, as predicted from Eqs. (2)–(4). However, in the HLC lens, the response time is determined by viscosity, wavefront bending speed, and active area of the photodetector. The obtained results show that the degrees of improvements in dynamic responses of LC cell (1.25× faster *τ*_*rise*_ and 9× faster *τ*_*fall*_) are different from those in dynamic responses of the HLC lens (8.5× faster focus time and 14× faster defocus time).

**Figure 7:**
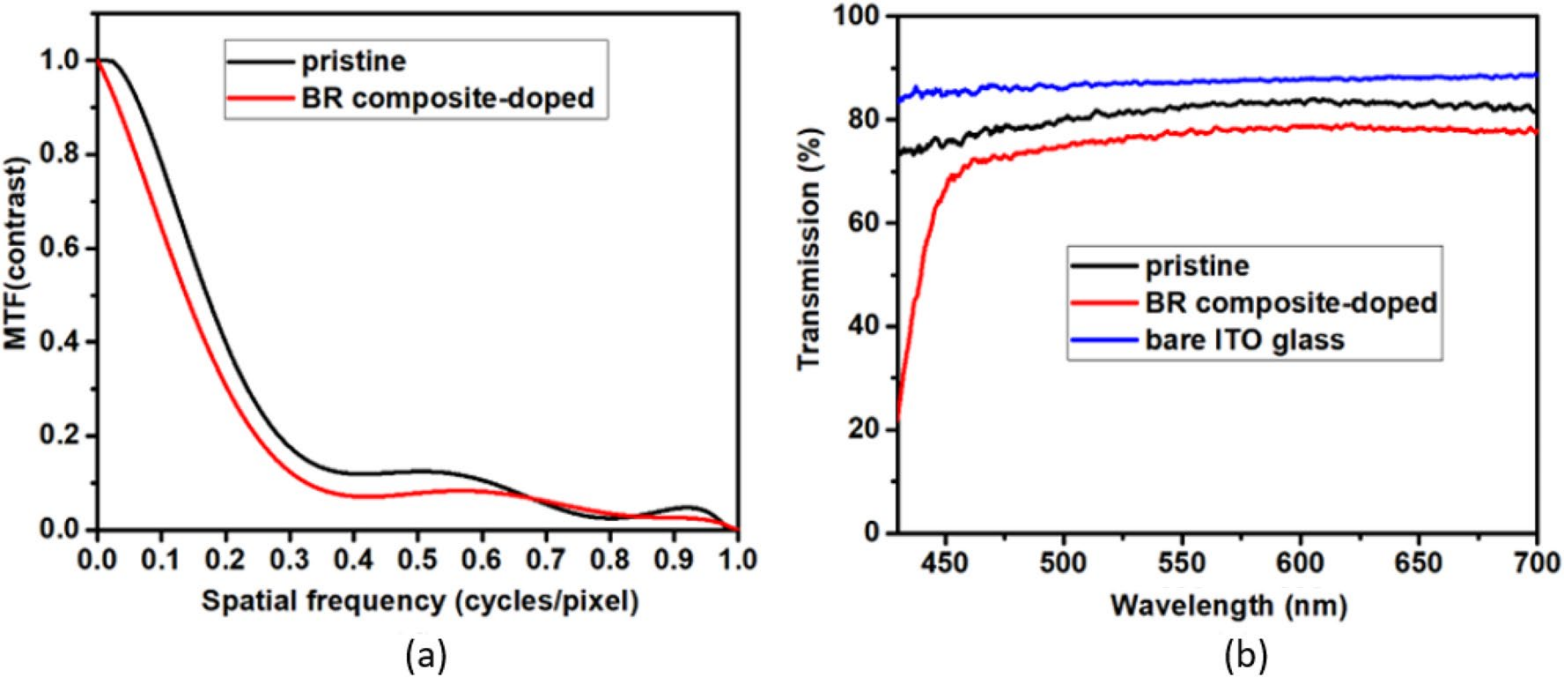
(**a**) MTFs of the images created through the pristine and BR composite-doped HLC lenses. (**e**) Transmission spectra of the bare ITO glass and the pristine and BR composite-doped HLC lenses.

The image qualities of the HLC lenses with maximum lens powers (MaxPs) were analyzed using the following setup. A toy object was placed in front of the HLC lens cell. A CCD camera with lens module was placed behind the HLC lens cell to capture formed image of object, where a polarizer was attached on the CCD camera with whose transmission axis parallel to the rubbing direction of the HLC lens cell. The distance between the CCD camera and HLC lens cell was set to 1 cm. The distance between the pristine HLC lens cell and the object was set to 28 cm, and that between the BR composite-doped HLC lens cell and the object was set to 37 cm, because these HLC lenses have different focal lengths. The modulation transfer functions (MTFs) of the captured images through the pristine and BR composite-doped HLC lenses were demonstrated with software Quick MTF Light 2. The MTF is defined as *(I*_*max*_ – *I*_*min*_)/(*I*_*max*_ + *I*_*min*_), where *I*_*max*_ and *I*_*min*_ are the maximum and minimum intensity, respectively. Generally, MTF declines from 1 to 0 with increasing spatial frequency. Figure 7a shows that the MTF of the BR composite-doped HLC lens is slightly worse than that of pristine HLC lens at spatial frequency below 0.7 cycles/pixel, due to the scattering from the refractive index mismatch between the interfaces by BR composite doping. As shown in Fig. 7b, the BR composite-doped lens cell has a lower transmission compared to the pristine HLC lens, due to light scattering resulted by BR composite. Notably, the BNA additive induces an intrinsic absorbance in wavelengths less than 450 nm^16^. The transmission of a bare ITO glass is also shown for reference.

## Conclusions

BR nanocomposite has been used to fabricate a fast response HLC lens. The BR composite-doped HLC lens provides a wider tunable focal range within smaller voltage interval (116.6–34.6 cm within 20–80 V) than the pristine LHLC lens (97.9–26.0 cm within 20–100 V). Moreover, the BR composite-doped HLC lens emerges a low RMS error of 0.06 λ at maximum lens power. Notably, the BR composite-doped HLC lens has the focus and defocus times that are 8.5× and 14× faster than the pristine HLC lens. Meanwhile, the focus and defocus times of the BR composite-doped HLC lens are less than 1 s. The fast focus and defocus responses are attributed to the decreased *γ* by SPEFs of the additives. The strengthened electric field surrounding TiO_2_ NP also assists in accelerating the focus response. The calculated time-dependent phase profiles of *γ*_235_- and *γ*_45_-HLC lenses confirm the decrease in defocus time is not simply proportional to the viscosity decrement. The detailed mechanism for the synergistic interactions between the BNA molecule and TiO_2_ NP is still under investigation.
